# PACAP and its role in primary headaches

**DOI:** 10.1186/s10194-018-0852-4

**Published:** 2018-03-09

**Authors:** Lars Edvinsson, János Tajti, Levente Szalárdy, László Vécsei

**Affiliations:** 1 Department of Medicine, Institute of Clinical Sciences, Lund University, 221 84 Lund, Sweden; 2 Department of Neurology, Faculty of Medicine, Albert Szent-Györgyi Clinical Center, University of Szeged, Semmelweis u. 6, Szeged, H-6725 Hungary; 3 MTA-SZTE Neuroscience Research Group, University of Szeged, Semmelweis u. 6, Szeged, H-6725 Hungary

**Keywords:** PACAP, CGRP, Receptors, Migraine, Cluster headache

## Abstract

Pituitary adenylate cyclase-activating peptide (PACAP) is a neuropeptide implicated in a wide range of functions, such as nociception and in primary headaches. Regarding its localization, PACAP has been observed in the sensory trigeminal ganglion (TG), in the parasympathetic sphenopalatine (SPG) and otic ganglia (OTG), and in the brainstem trigeminocervical complex. Immunohistochemistry has shown PACAP-38 in numerous cell bodies of SPG/OTG, co-stored with vasoactive intestinal peptide (VIP), nitric oxide synthase (NOS) and, to a minor degree, with choline acetyltransferase. PACAP has in addition been found in a subpopulation of calcitonin gene-related peptide (CGRP)-immunoreactive cells in the trigeminal system. The PACAP/VIP receptors (PAC_1_, VPAC_1_, and VPAC_2_) are present in sensory neurons and in vascular smooth muscle related to the trigeminovascular system. It is postulated that PACAP is involved in nociception. In support, abolishment of PACAP synthesis or reception leads to diminished pain responses, whereas systemic PACAP-38 infusion triggers pain behavior in animals and delayed migraine-like attacks in migraine patients without marked vasodilatory effects. In addition, increased plasma levels have been documented in acute migraine attacks and in cluster headache, in accordance with findings in experimental models of trigeminal activation. This suggest that the activation of the trigeminal system may result in elevated venous levels of PACAP, a change that can be reduced when headache is treated. The data presented in this review indicate that PACAP and its receptors may be promising targets for migraine therapeutics.

## Background

The field of PACAP and its receptor PAC_1_ is currently very topical in the migraine field. Here we have provided an overview of available data bases; we used PubMed, Medline and Google Schoolar as sources. We report that PACAP is released in conjunction of migraine and cluster headache attacks. The most likely source of PACAP contributing to this elevation in cranial outflow resides in the trigeminovascular system (TVS) and the parasympathetic otic and sphenopalatine ganglia. The PAC_1_ receptor is less well described in the TVS, but found in the trigeminal and sphenopalatine ganglia. It is hypothesized that this may be a second neuropeptide system that might be useful to block in acute attacks.

### Introduction

Migraine is thought to involve a primary dysregulation of sensory processing in the central nervous system (CNS) as a starting point of migraine [1]. It is thought that the origin of the disease rests in the hypothalamus – thalamus system, coupling to the brainstem. The central involvement is reflected early in many patients experiencing tactile allodynia and other neurological symptoms that affect the senses, including sensitivity to light, sound, and smell. These are thought to be consequences of central sensitization within the trigemino-thalamic pathway. The trigeminovascular system is involved with all its parts; the trigeminal nerve, the cranial vasculature, and nuclei within the brainstem/spinal cord.

Pituitary adenylate cyclase-activating polypeptide (PACAP) and vasoactive intestinal peptide (VIP) are members of the VIP/secretin/growth hormone-releasing hormone/glucagon neuropeptide superfamily, widely expressed in vertebrate tissues [2–6]. VIP was first described by Victor Mutt during the early 1970s [7]. It was isolated from pig intestine and is localized to most organs and tissues in the body. During the following years, the other members of the family were described and characterized, including peptide histidine isoleucine/methionine (PHI/PHM), secretin, and PACAP [3, 8, 9]. The PACAP neuropeptide was discovered nearly 30 years ago based on its ability to enhance adenylate cyclase activity in rat pituitary cells, and was first isolated from ovine hypothalamic extracts [10]. Numerous studies on the localization/distribution/release of this group of neuropeptides from different tissues and involvement in diseases have been presented [11, 12]. The aim of this review is to focus on sensory aspects of PACAP and primary headaches, with special emphasis on migraine.

### General features and localization of PACAP within the CNS

There are two forms of PACAP (− 27 and − 38), with both isoforms sharing functional and structural homologies with VIP [10] and being able to bind to G-protein-coupled receptors specific (PAC_1_) and less specific (VPAC_1_ and VPAC_2_; with comparable affinity to VIP, PACAP-27, and PACAP-38) to PACAP [3, 13, 14]. Recently, however, the functional presence of yet unidentified PACAP receptors have been proposed based on experimental results using selective modulators [15].

PACAP is a pleiotropic peptide that can be found in several exocrine and endocrine tissues, in the respiratory, intestinal, urinary, and reproductive systems, as well as in the central and peripheral nervous systems [16–23]. In neuronal tissues, the isoform PACAP-38 predominates. It is known to play hypophysiotropic, neuromodulatory, and neurotransmitter roles, and has been associated with differentiation- and proliferation-inducing effects in the developing nervous system, as well as with cytoprotective, anti-apoptotic, and anti-inflammatory features within various target organs [24–34]. Furthermore, PACAP has been shown to be a potent local and systemic vasodilator administered in a wide concentration range, associating with biphasic paradoxical hypertensive effects in very large doses [35–37].

**Fig. 1:**
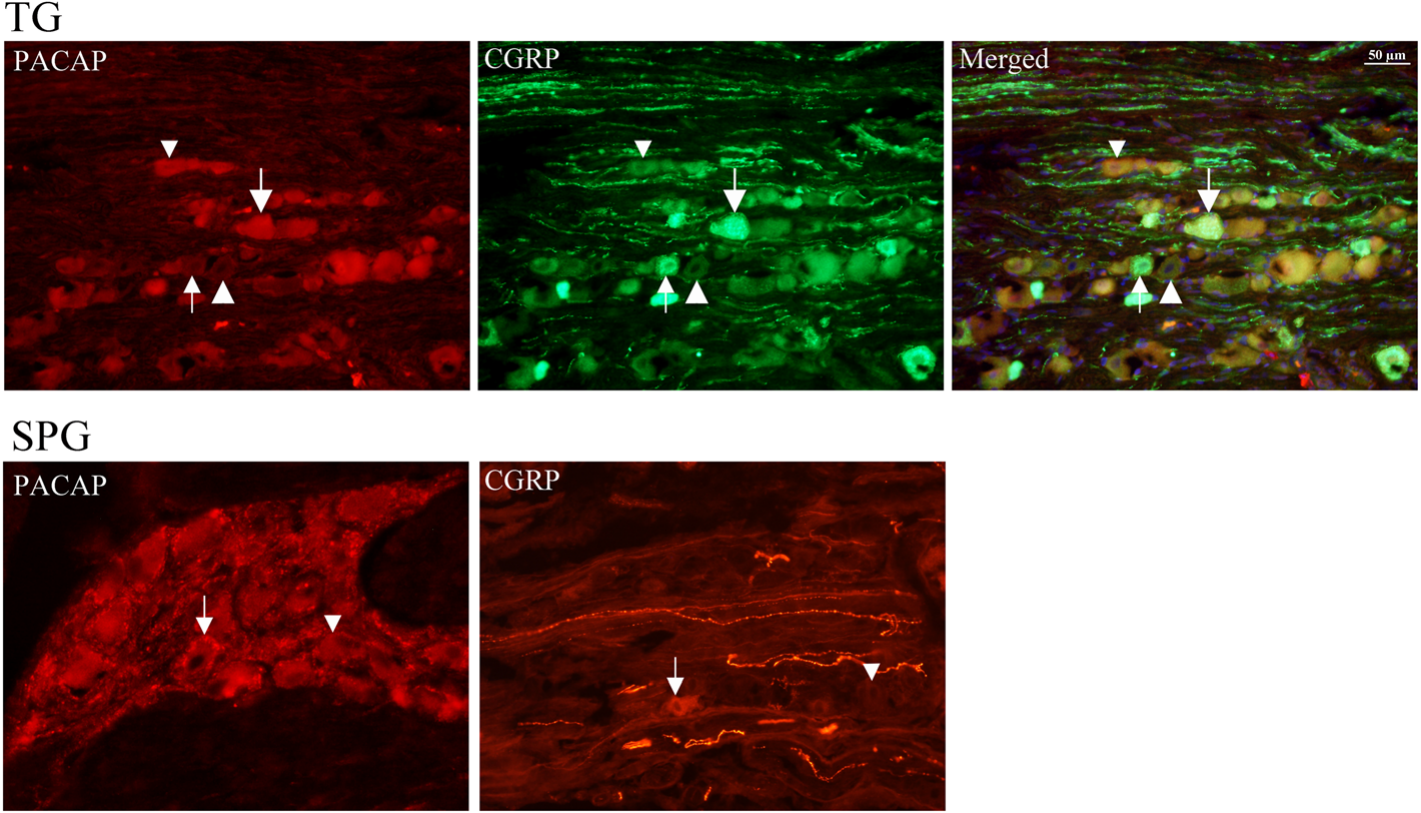
Immunohistochemical demonstration of distribution of PACAP and CGRP in the sphenopalatine ganglion (SPG) and the trigeminal ganglion (TG). The figures illustrates that in both ganglia there are cells storing the two neuropeptides and also being alone. The CGRP immunoreactivity occurs more frequent in the TG than does PACAP-ir. Arrows show CGRP positive and PACAP negative cells, while arrow heads show co-localization (above) or negative (below). There are also fibers positive to CGRP. In the SPG the arrow shows a single CGRP positive cell that co-localize PACAP while numerous cells are positive to PACAP only (arrow head). In the right hand SPG illustration  there are several CGRP positive fibers

Immunohistochemical evidence confirms the presence of PACAP in anatomical structures relevant to the pathogenesis of primary headaches including migraine, such as in the trigeminocervical system, including nerve fibers in the dura mater, the cerebral vessels [38], the facial skin [39], the trigeminal ganglion (TG) [15, 39–44], the trigeminal nucleus caudalis (TNC) [15, 16, 45], the Rexed 1 and 2 laminae at the C1-C2 levels of the cervical spinal cord [45] and in various brainstem nuclei within the ‘migraine generator area’ [16, 46, 47] as well as in the parasympathetic sphenopalatine (SPG) and otic ganglia (OTG) [40, 41, 48–50]. Recent works have revealed that PACAP is localized in small neurons in the dorsal root ganglia and the TG which in addition store calcitonin gene-related peptide (CGRP) [44]. Interestingly PACAP mRNA-labeled neurons constituted 10% of the total number of nerve cell bodies in the dorsal root ganglia, whereas the population of CGRP mRNA-labeled nerve cell bodies constituted 46% [51]. This agrees well with later in depth immunocytochemistry work on the TG [52] (Fig. 1).

In line with the above, early studies revealed the presence of specific PACAP-binding sites in relevant regions of the rat and human CNS [53, 54]. In addition, subsequent immunohistochemical and biochemical evidence demonstrated the presence of PAC_**1**_ and VPAC_**2**_ receptors in the rat TG [55]. Of note, there exist some discordant results, functional [56] and biochemical data on the mRNA level suggest that VPAC_**1**_ receptors are not present in the TG of rats [55]. However, mRNA for all three types of VIP/PACAP receptors have been reported to occur in human and rat TG [57], and this is supported by another study in rat TG and TNC [15]. Moreover, protein expression has been shown by immunohistochemistry for all three types of PACAP receptors in both the human and rat SPG [49]. The evidence suggest a special role in terms of disease pathogenesis among relevant neuropeptides. The detailed histochemistry revealed that while VIP/PACAP are both seen in the parasympathetic ganglia (SPG/OTG), only PACAP has been found in the trigeminal system [42].

### Early findings of neuropeptide release in primary headaches

The role of PACAP and VIP is under discussion because (i) they appear jointly in most of the SPG/OTG neurons and (ii) PACAP co-localizes with CGRP only in a small fraction of neurons within the TG in the small to medium sized neurons. Early studies showed that in migraine VIP was only released in a subpopulation of patients that had facial symptoms such as nasal congestion, rhinorrhea, and scleral injection in addition to headache (probably due to the activation of the parasympathetic system). Importantly, all cluster headache subjects showed both facial symptoms and VIP release [58]; however, at that time, the methodology to measure PACAP was not available. We suggested that this was due to activation of the parasympathetic system. Experimental studies in cats [59] revealed that the activation of the trigeminal system may in addition involve the parasympathetic system, as denervation of the trigeminal nerve aborted the activation of SPG, interpreted either as a direct or indirect coupling between these two systems.

### Involvement of PACAP in migraine

#### Conclusions from the studies with PACAP infusion and knockouts

Human provocation studies of migraine using systemic administration of different molecules have generated important insights into the possible mechanisms in primary headache diseases. CGRP, VIP, and PACAP-38 are all strong vasodilators, sharing the activation of adenylate cyclase. The first direct evidence that highlighted the potential significance of PACAP in the pathophysiology of migraine came from observations made in a human study [60] where intravenous administration of PACAP-38 caused headache and vasodilation in healthy subjects as well as in migraineurs, and lead to delayed-type migraine-like attacks exclusively in migraine patients without aura (65–75%), a feature remarkably similar to that previously reported after nitroglycerin infusion [61, 62]. Because VIP had no such effect, it was suggested that this might be secondary to the activation of the PAC_1_ receptor, which is now under study in a clinical trial. For comparison, CGRP induces delayed migraine-like attacks in 65% of migraine patients, but not in normal subjects [63]. Today numerous CGRP active agents are on the verge of going to the market as effective therapy both for acute treatment and as prophylaxis.

In support of the above, a magnetic resonance imaging (MRI) angiographic study revealed that PACAP-38-induced headache was associated with significant dilation of the extracranial part of the middle meningeal arteries (MMAs), but not of the middle cerebral arteries (MCAs). This effect preceded the onset of migraine-like headache and was sensitive to the administration of a triptan class of drug [64]. A subsequent MRI angiographic human study demonstrated a remarkable and sustained vasodilation of extracranial but not intracranial arteries (including the MCAs) following PACAP-38 administration [65]. In addition to these, intravenous PACAP-38-induced migraine-like attacks have been found associated with alterations in brain network connectivity, by means of a resting-state functional MRI study [66]. The role of VIP in PACAP-induced vasodilation is unclear, as data regarding its levels after PACAP administration are contradictory [65, 67], and its vasodilation is less long-lasting than that of PACAP [65]. The migraine response to PACAP-38 has not been found to be influenced by the presence of the risk allele MEF2D [67, 68].

Experimental studies support the relevance of PACAP in sensory functions as well as in pain disorders, including primary headaches. Neurograms obtained following stimulation of the ipsilateral peroneal or sciatic nerve suggested that intrathecal PACAP may act as a neurotransmitter or modulator in sensory C-fibers, possibly with a contribution of A-fibers [69]. Interestingly, PACAP-27 was more potent than PACAP-38 or VIP at this function. On the other hand, the direct intrathecal administration of PACAP elicited a dose-dependent decrease in the tail-flick latency, whereas higher doses resulted in biting and scratching; these behaviors were interpreted as pain-like syndrome and supported a role of PACAP as a sensory neurotransmitter involved in nociception [70]. Intrathecal administration of PACAP(6–38), a PAC_1_ receptor antagonist reduced mechanical and thermal hyperalgesia, suggesting that these receptors are important for PACAP-induced pain [71]. The role of PAC_1_ receptor in pain sensation has also been supported by studies using PAC_1_ knockout mice, as these animals showed reduced chronic responses to chemical, mechanical, and thermal stimuli [72, 73]. Similar results were obtained with PACAP-knockout mice, presenting with diminished light-aversive behavior (i.e., photophobia), decreased c-fos expression in the TNC and TG, and reduced meningeal blood flow following nitroglycerin administration compared to wild-type controls [74].

PACAP has also been implicated in higher order processing of pain in brain regions such as thalamus and amygdala [75, 76], relevant in the central sensitization and emotional load of pain. Intracerebroventricularly applied PAC_**1**_ receptor inhibitor decreased the delayed activation and sensitization of second-order nociceptive neurons within the trigeminocervical complex following dural stimulation [77]. This supports a potential role of PACAP in the development of central sensitization of pain in migraine. There are experimental results suggesting the role of PACAP in the development of peripheral sensitization as well in certain models of pain [78]. The data on the possible role of PACAP and its receptors in migraine pathogenesis by means of modulating neurogenic inflammation [73, 79–81] and mast cell degranulation [65, 82–86] are, however, conflicting.

#### PACAP as a biomarker in primary headaches

A support for a sensory role of PACAP in nociception came from experiments with capsaicin administration in vivo in rats [26], as it resulted in elevated cerebrospinal fluid concentrations of PACAP-27 by 177%, PACAP-38 by 93%, and CGRP by 692% [87]. Studies on animal models of trigeminal activation confirm the potential role of PACAP in the pathogenesis of migraine in particular, as immunoreactivity of both PACAP-27 and PACAP-38 were found elevated in the TNC of rats following electrical stimulation (at the TG) or chemical stimulation (by nitroglycerin) of the trigeminovascular system [88]. Furthermore, PACAP-38 levels were found elevated in the blood plasma of rats after electrical stimulation of the TG [88]. Likewise, elevated levels of PACAP were measured in the external jugular vein samples of cats following electrical stimulation of the superior sagittal sinus [89]. In fact, stimulation of the trigeminal system directly showed the release of not only PACAP but also VIP and CGRP into the external jugular venous blood [89, 90].

Human clinical studies provide concordant data, as of note, elevated levels of plasma PACAP-38 were revealed in the ictal period of migraineurs (i.e., during a spontaneous migraine attack) compared to the interictal phase [89, 91]. Interestingly, supportive data were reported in episodic cluster headache patients as well [92]. The interictal plasma PACAP concentrations of migraineurs were, however, significantly lower compared to the levels measured in healthy controls or in patients with tension-type headache [89, 91, 93].

### Concluding remarks

The exact role of PACAP in primary headaches is still unknown but it is clearly present in many different parts of the CNS including several regions of interest in the discussions of migraine pathophysiology. Current evidence suggest that PACAP plays essential roles in the pathogenesis of migraine via modulating the function of nociceptive neurons within the trigeminocervical system.

The position of PACAP in migraine rests on the following main observations: (i) PACAP levels are increased in the circulation of cats and rats upon stimulation of the superior sagittal sinus [89, 94], and the TG, respectively [88]. (ii) PACAP levels in the external jugular vein are reduced with amelioration of migraine headache when subjects were treated with sumatriptan, and lower levels of PACAP occur between attacks when compared with attacks [89]. (iii) Systemic infusion of PACAP-38 in migraine patients results in migraine-like headache [60]. (iv) The plasma levels of PACAP-38 are altered in ictal and interictal periods of migraine patients [91]. The unanswered questions that remain are related to the origin of endogenous PACAP and the mechanism through which systemic PACAP can induce migraine. Though Banks suggests that PACAP has actions in the CNS and provides evidence in support [95], the available data strongly indicate that PACAP does not pass the wall of cerebral arteries, as studied in isolated perfused MCAs [96], which agrees with the view that the peptide is a large molecule that does not pass the blood-brain barrier (BBB) [96, 97]. Therefore, as suggested also for CGRP, the relevant target of systemically administered PACAP could very well be effects on sites located outside the BBB, such as the TG and other ganglia as well as the dura mater; however, this is still not settled, and the role of the BBB needs to be clarified.

## Conclusions

1. The concordant experimental and clinical data show that PACAP and its receptors may have a role in the pathogenesis of primary headaches, especially migraine and cluster headache.
2. The relation with other neuronal messenger molecules deserves future study.
3. Collectively the current understanding of PACAP and its receptors in relation intracranial structures and brain provide valuable information as potential targets of effective future therapeutics.
